# Achieving *inclusive* research priority-setting: what do people with lived experience and the public think is essential?

**DOI:** 10.1186/s12910-021-00685-5

**Published:** 2021-09-04

**Authors:** Bridget Pratt

**Affiliations:** Queensland Bioethics Centre, Australian Catholic University, 1100 Nudgee Road, Banyo, QLD 4014 Australia

**Keywords:** Ethics, Inclusion, Power, Priority-setting, Engagement, Partnership, Health research, Patient and public involvement

## Abstract

**Background:**

Engagement of people with lived experience and members of the public is an ethically and scientifically essential component of health research. Authentic engagement means they are involved as full partners in research projects. Yet engagement as partnership is uncommon in practice, especially during priority-setting for research projects. What is needed for agenda-setting to be shared by researchers and people with lived experience and/or members of the public (or organisations representing them)? At present, little ethical guidance exists on this matter, particularly that which has been informed by the perspectives of people with lived experience and members of the public. This article provides initial evidence about what they think are essential foundations and barriers to shared decision-making in health research priority-setting and health research more broadly.

**Methods:**

An exploratory, qualitative study was conducted in 2019. 22 semi-structured interviews were performed with key informants from the UK and Australia.

**Results:**

Three main types of foundations were thought to be essential to have in place *before* shared decision-making can occur in health research priority-setting: relational, environmental, and personal. Collectively, the three types of foundations addressed many (but not all) of the barriers to power sharing identified by interviewees.

**Conclusions:**

Based on study findings, suggestions are made for what researchers, engagement practitioners, research institutions, and funders should do in their policy and practice to support meaningful engagement. Finally, key international research ethics guidelines on community engagement are considered in light of study findings.

## Introduction

Patient and public engagement is gaining prominence in health research, with research institutions, international research ethics guidelines, and funding bodies now promoting, or even mandating, engagement as an ethically and scientifically essential component of all health research [8, 28, 36, 39]. Authentic engagement means involving patients, members of the public, or organisations representing them as full partners or collaborators [46]. This implies shared decision-making throughout projects and the greatest community empowerment [37]. It entails involving individuals with lived experience, members of the public, and/or organisations representing them as decision-makers not only when conducting data collection and analysis and disseminating findings *but also* when setting research projects’ topics and questions and shaping projects’ design [13, 36, 43]. In this paper, the terms ‘people with lived experience’ and ‘members of the public’ are primarily used rather than patient, community, or community member. These terms are used because they capture two key perspectives that people who are engaged bring to research studies: (1) the lay/public/citizen perspective and (2) the patient/community/service user perspective.^1^

Engaging people with lived experience and/or members of the public, especially those from groups who are considered disadvantaged or marginalised by social institutions and norms, as collaborators is essential as a matter of justice [27, 33, 34]. It provides a path for making their voices and concerns more visible in *agenda-setting* and in the production of scientific knowledge [36]. It can help address epistemic injustices and generate research project topics and questions that are more explicitly focused on improving access and affordability of health care and services for them [2, 27, 33, 34]. Thus, their involvement in priority-setting is especially important.^2^

Yet it is uncommon for such individuals, especially those considered disadvantaged or marginalised, or organisations to be included as decision-makers during agenda-setting for research projects. They rarely initiate new research projects or are invited by researchers to have a say in setting agendas or designing research projects. A recent quantitative study found that over 60% of community organisation respondents had rarely or never jointly submitted a grant application when working in collaborative research partnerships [40]. Current funding mechanisms make it difficult to undertake engagement pre-grant award. Indeed, even when invited to enter decision-making spaces, unequal power dynamics between researchers and people with lived experience and/or members of the public can give rise to tokenism in priority-setting: presence without voice and voice without influence, particularly for the most marginalised [38].

What is needed for priority-setting to be shared by researchers and people with lived experience and/or members of the public (or organisations representing them)? At present, little ethical guidance exists on this matter, particularly that which has been informed by the perspectives of people with lived experience and members of the public. A significant amount of existing literature explores the concepts of engagement and participation in contexts of power disparities, spanning disciplines like political philosophy (see [4, 47, 48]), development studies (see [3, 7, 9, 12, 14, 15]), health policy (see [1, 22, 23]), and community-based participatory research (see [6, 19, 24, 31, 45]). That body of work largely does not consider engagement in the context of research priority-setting [35]. Within it, there is also limited literature drawing on the voices and perspectives of people with lived experience, members of the public, and engagement practitioners.

This study aimed to access their voices to investigate what is essential for shared decision-making during agenda-setting for health research projects. Robust ethical guidance is informed by both theory and the considered judgements of relevant persons—in this case, those with key insights and experiences of engagement in health research. This encompasses not only researchers but also people with lived experience, engagement practitioners, and members of the public. If the latter voices aren’t captured, they are largely absent from ethics discourse and a key source of information is excluded or missing. Talking with them about power sharing in health research addresses an epistemic injustice and helps democratise knowledge within the ethics field.

22 semi-structured interviews were conducted with key informants from the UK and Australia. Both Australian and UK participants were included to explore this topic because engagement in health research is established in both countries. Since “patient and public involvement” in research has been a feature of UK policy for longer and is widely adopted by UK research funders,^3^ it was thought that UK interviewees might have different ideas and experiences related to power-sharing than Australian interviewees. Data were thematically analysed and insights regarding what foundations are needed and what barriers exist to shared decision-making in health research are reported. Foundations and barriers refer to factors that can facilitate or obstruct power-sharing; they help create conditions where power-sharing can or or are unlikely to occur. Foundations are necessary for power-sharing to happen; barriers hinder its occurrence.

The paper then critically reflects on the key lessons this study offers for sharing power with people with lived experience and members of the public in health research *priority-setting*. Based on study findings, suggestions are made for what researchers, engagement practitioners, research institutions, and funders should do in their policy and practice to support inclusive research priority-setting. Their specific ethical responsibilities are proposed because their remits make them especially well-placed to build certain foundations. Finally, key international research ethics guidelines on community engagement are considered in light of study findings.

## Methods

### Study methods and sample

In-depth interviews were chosen as the primary method to explore the topic because they allow for the rich details of key informants’ experiences and perspectives to be gathered. 22 semi-structured interviews were conducted with key informants in three main categories:

- People with lived experience who are or have been involved in health research (16)
- Members of public who are or have been involved in health research (2)
- Engagement practitioners who work in health research (4)

Engagement practitioners are individuals who work for research institutions and their role is to support and train researchers to engage patients and the public in research projects; build relationships between researchers, research institutions, and the public; and build patient and public capacity to engage in research.

Sampling was initially purposive; potential participants with lived experience who had been involved in health research and engagement practitioners were identified in the UK and Australia through BP’s existing networks. In Australia, snowball sampling and posting information about the study on the Research4Me^4^ Facebook group were then used to identify additional interviewees. In the UK, information about the study was sent out on a university’s patient and public involvement email listserv and this generated the remainder of interviewees.

In total, five men and seventeen women were interviewed. Twelve interviewees live in the UK and ten in Australia. Interviewees had lived experience of mental health conditions (2), chronic illness (6), and forms of disability (physical, psychosocial, cognitive) (6). Two interviewees with lived experience did not disclose the condition(s) with which they were living. Nine interviewees (eight with lived experience and one member of the public) had experience with research priority-setting; two were from Australia and seven were from the UK. Interviewees’ engagement experiences ranged from being a single, short-term engagement (e.g., one focus group) to being engaged in one or more research projects over one to five years to having decades of engagement experience over many projects. Interviews continued until data saturation was achieved.

### Data collection and analysis

During interview, people with lived experience and members of the public were first asked what roles they had been engaged to perform in health research. Subsequent interview questions asked about their perspectives and experiences sharing power in the context of that or those specific role(s). This was because not all participants had experience in co-design of research projects, which entails being engaged during agenda-setting, or in research priority-setting.^5^ Where interviewees had a priority-setting role, interview questions were asked in the context of that role only. Collectively, interviewees had the following roles in health research: member of funding panel, member of priority-setting process for the James Lind Alliance, co-applicant, community researcher, member of steering or advisory group, and/or member of focus group. Engagement practitioners were asked about their experiences and perspectives on co-design. Thus, the study data speak to power-sharing not only in health research priority-setting but also more broadly.

Interviews were transcribed verbatim and thematic analysis was undertaken by two coders in the following five phases: initial coding framework creation, coding, inter-coder reliability and agreement assessment, coding framework modification, and final coding of entire dataset [5, 16]. The initial coding framework was developed by BP and NE co-coding five transcripts from Australian interviewees independently and jointly coming up with a list of codes. The remaining Australian interviews were then coded by BP and the initial list of codes was revised. Using the initial coding framework, BP and JS next undertook an iterative process of coding a UK interviewee transcript, assessing intercoder reliability and agreement, and modifying the coding framework [16]. A second co-coder (JS) was brought in to see if the coding framework could be reliably applied by someone with no prior involvement in the study and to test that the coding framework was applicable to the UK interviewee data. Six transcripts were co-coded and 100% intercoder agreement was achieved, with agreement going the way of both parties fairly evenly in most cases. Fifteen new subcategories (of 61 subcategories total) were added to the coding framework based on the UK data. Once the coding framework was finalized, BP applied it to recode all 22 transcripts. According to Campbell et al. [5], once high intercoder agreement is reached, a single person can perform the remaining coding, provided it is the person whose coding generally carried the day during the negotiation process.

### Study limitations

It is critical to acknowledge the main limitations of this study. First, interviewees were recruited from Australia and the UK only. While engagement in health research is increasingly common in both countries, there are other countries where engagement is frequently occurring in health research, including in low and middle-income countries. Future research should capture their views as well. The author has started to do so as part of two case studies of health research priority-setting being conducted in India and the Philippines.

The interviewee sample had fewer men than women, members of the public than people with lived experience, and individuals living in urban than rural areas. The diversity of interviewees is also somewhat unclear, as the study did not collect demographic data about interviewees. UK interviewees self-selected themselves to participate after information about the study was sent out on a university’s patient and public involvement listserv. That listserv in itself was not thought to be exceptionally diverse by the engagement practitioner who runs it. Lack of diversity was identified as a problem for engagement in health research as a whole by interviewees. Nonetheless, interviewees had lived experience of a range of disabilities (cognitive, psychosocial, physical) and chronic illnesses. Several mentioned being of non-Caucasian ethnicities such as African, Hungarian, and Indigenous. In terms of age, Australian interviewees spanned younger ages (20s and 30s) to retirement age. UK interviewees were generally older but not all were retired.

Finally, not all of interviewees’ insights were directly about priority-setting because they had not had roles during early phases in health research. Nearly half the interviewees had some priority-setting experience. More of these were from the UK, where engagement roles on funders’ grant panels and as co-applicants are more common.

## Results

### Foundations

Three main types of foundations were identified as essential to have in place *before* power sharing and meaningful engagement can occur in health research: relational, environmental, and personal. No differences were found between the foundations reported by Australian versus UK interviewees, though the UK research funding environment was described as more supportive of involving people with lived experience and members of the public in health research agenda-setting relative to the Australian research funding environment.

#### Relational

Two relational foundations were described: *forming connections* and *building trust*. Key types of connections to form are: (1) personal connections between researchers and the specific individuals who are engaged in research projects and (2) connections between researchers, their institutions, and the community or public.

Building *personal connections* entails having empathy, being open and honest, sharing personal information and stories, listening, putting one’s selves in each other’s shoes to understand where the other is coming from, learning each other’s strengths and weaknesses, and performing acts of kindness. Developing such connections is essential to inclusion:

Once you’ve developed that [relational understanding] between a gatekeeper to a society and someone that’s experienced oppression, you give them an olive branch to become included. So that to me is key. (person with lived experience, Australia)

Building *connections with the community or public* were described as important to sharing power in agenda-setting:

I do see good researchers do that, you know they, they will spend a couple of years mingling with a community before they ask for something. I think it’s really good practice. (person with lived experience, Australia)

Making ourselves as organisations and as researchers accessible and not asking people to step over our thresholds but stepping over our own thresholds to go and really be accessible to others. (engagement practitioner, UK)

Such connections are necessary for building trust and for building awareness of engagement in research, its value, and the relevance of research to people’s everyday lives.

*Informal interactions* were identified as key to forming both types of connections. Interviewees noted that taking the time to do less research-focused activities at the outset is just as important as moving on to co-design, particularly when engaging those with experience of marginalisation. To build personal connections, creative activities like crafting or harmonica lessons were described by a UK engagement practitioner as “especially levelling” because “if you can learn to do something with someone where you're both equally unknowledgeable and unpractised, it creates a bond to start you off.” To build connections with communities, activities like community festivals, film nights, and introductory/education sessions with panels that combine researchers and people with lived experience were discussed. Such activities largely:

Had nothing to do with sitting around the table doing co-design with our researchers, but they also had everything to do with how we build our relationship with our community that leads to people wanting to come and sit at the table with us… we set up an expectation that what we really value is our difference of opinion… we are respectful of all voices and we wanna hear all voices. (engagement practitioner, Australia)

Building trust was also identified as a core foundation of power sharing. Trust ran two ways. Researchers needed to have trust in those they had engaged; this was discussed as people with lived experience and members of the public having “credibility”:

You are the consumer, you are the outsider, you have to prove that you’re up to this and then if you manage to establish your credibility people will suddenly start listening to you. (person with lived experience, Australia)

People with lived experience and members of the public had to have trust in researchers and the institutions for whom they work in order to share power with them. According to an Australian engagement practitioner:

When you’ve got that basis of trust there, you can sit across the table and then go actually I really disagree with that you’re saying, what I want you to hear from me is this and you can have far more robust, equal power sharing relationships.

Here, s/he refers to relationships throughout the research process, from priority-setting onwards.

Building these connections and trust makes people who are engaged in health research feel comfortable sharing their vulnerabilities and being critical with researchers. Although people’s vulnerabilities are the hardest to draw out, these are the key stories for agenda setting. Researchers can identify those problems that pain people the most and pursue them in research.

#### Environmental

Three environmental foundations were described: *researcher support*, *funding mechanisms and policies,* and *norms*. Each must be present in the research environment in order for power sharing to occur in priority-setting. Researcher support meant researchers provide resources and create spaces that enable people with lived experience and members of the public to participate, so that “people who are less confident, that are coming from a marginalised position, can step powerfully into that [research decision making] space.” Five key supports were identified:

- Training for those engaged (and for researchers)
- Accommodating varying needs
- Creating a safe space to share vulnerabilities and be critical
- Pairing or mentoring system
- Making people feel valued

Researchers should provide training for those engaged in health research based on their needs. Sixteen interviewees (including four engagement practitioners) noted the importance of training for those engaged. This would encompass training about grant writing and funding processes; ethics processes; research processes, methods, and jargon; and patient and public involvement roles in research. For agenda-setting, training about grant writing and funding processes were identified as especially key by a person with lived experience from Australia:

Skills around okay well what does grant writing look like… a lot of that stuff is really kind of like university bureaucratic behind the scenes stuff and that’s really like where the power kind of relations really are… I also was interested in those processes but that’s kind of not available to you as a community researcher sometimes I think.

Training around grant writing and funding processes is often not accessible to people with lived experience or members of the public and excludes them from participating in those phases of research projects.

Training for researchers was also discussed and entailed introducing them to what patient and public engagement is and how to undertake it in a way that is genuinely inclusive. To start the training process, a UK engagement practitioner noted that s/he might:

have a panel of which the researchers can, who come, can post their questions and we’ll also have some sort of pre-decided questions so that patients can talk a bit about their experience and then we’ve got some group work. And it’s, as much as anything, I think it’s trying to show researchers that a lot of researchers who don’t see patients day to day are quite frightened of them, they’re really anxious about talking to patients and I think it’s showing them that they aren’t there unnecessarily to criticise, patients really, genuinely, they really want to help and they will do that in a constructive way.

Researchers should accommodate varying needs, which was defined by people with lived experience as making reasonable adjustments so engagement activities can be accessible and performed and as supporting in “an unequal manner to provide equity”. Two ways of accommodating were spoken about (and overlap with each other): providing information in ways those engaged can understand and taking account of disabilities (physical (mobility, vision, hearing), psychosocial, cognitive). The former included (but was not limited to) writing for varying literacy levels and language fluencies, not using technical jargon, and having language interpreters at meetings. The latter included (but was not limited to) making things physically accessible, using easy to read large print, employing sign language interpreters, and being flexible about how tasks could be completed by those engaged.

Researchers should also create safe spaces for engagement, make those engaged feel valued, and employ a pairing or mentoring system where possible. A safe space was described as an atmosphere where people are encouraged to be critical and where the people are comfortable with each other. They feel free to speak up and share their experiences and vulnerabilities with the group without feeling “stupid”. Making people feel valued was discussed by a UK person with lived experience as providing a comfortable venue with refreshments, remembering people’s names and things about them, being friendly and welcoming, and making statements such as “we really value [you] and feel you can make a valuable contribution”. A pairing system could link a person with little to no engagement experience with another person with significant engagement experience. It could also pair someone with lived experience or from the public who had been engaged with a researcher.

Funders should offer funding for pre-grant engagement, which supports people with lived experience and members of public to be part of developing grant applications and setting research project agendas:

In England, there’s local organisation so they, they operate across an area called research design service, RDS, and they can give researchers access to some pots of money that can help them do the patient and public involvement work before the main funding comes, if it comes. As I say in Scotland that doesn’t exist I’ve been told. (person with lived experience, UK)

They should make engagement a funding criterion too, which some funders have done. According to a UK interviewee with lived experience, the UK National Institute of Health Research has such criteria. S/he states:

If you don’t show evidence that you’ve actively involved people with a condition, then you’ve got no chance whatsoever getting funded [by the National Institute of Health Research].

These supports and funding practices are valuable because they enable people with lived experience and members of the public to be engaged early in health research (pre-grant award) and feel comfortable sharing their stories and their criticisms of proposed research.

Beyond research support and funding, an environment is needed where the norms surrounding research and public engagement support power sharing in health research. Where research culture values different forms of experience and evidence, it can facilitate community knowledge being valued and used in agenda-setting and design of health research projects.

#### Personal

Specific personal qualities and skills of researchers and of people with lived experience and members of the public were identified as essential pre-requisites for power sharing in health research. Lead researchers who value engagement were strongly identified as essential to share power throughout research, including during priority-setting:

I have heard from people who have done a lot of PPI [patient and public involvement], when they get, feel like the chief or the principal investigator is fully onboard with it and treats them as like you know an equal and bothers to keep in touch with them, that is absolutely vital. (engagement practitioner, UK)

Aside from valuing engagement and co-design, other qualities that researchers ideally should have are being humble and being open to sharing personal information to develop relationships and to listening to people who have different opinions to them. Essential skills were good communication, facilitating engagement and co-design, negotiation, and conflict resolution.

The qualities and skills people with lived experience and members of the public who are engaged in health research should include that they:

- Reflect the diversity of lived experience of using a service or a community
- Are well-connected and informed across the community: Have deep understanding of their community and the issues impacting it
- Can be a voice for others and tell their stories; Aren’t fixated on their own problems
- Want to make a difference for others and the health system
- Are confident to speak up and be assertive
- Are articulate
- Are credible^6^
- Have analytical skills
- Have team-work and interpersonal skills
- Have negotiation and conflict resolution skills

Selecting people with lived experience and members of the public with the confidence to sit at a table with senior researchers and to challenge their ideas was identified as critical where researchers have limited experience with engagement and co-design and/or have a tokenistic view of what it entails. According to an Australian engagement practitioner,

it felt like this first experience of co-design was a test case that was kind of winning them over to a new way of working. So for this particular co-design, especially I needed really skilled people sitting at the table coming from a lived experience perspective because it’s cracking open the door and opening the way.

Understanding research and having credibility could also be useful qualities to have in that context. However, the interviewee also recognised that selecting confident and articulate individuals could easily exclude voices. A research institution’s engagement program should thus seek to build people’s capacity to engage in research for a diversity of individuals within the given community or public “so that people that are less confident, that are coming from a more marginalised position can step up into that space.”

### Barriers

Seventeen barriers to sharing power in health research were identified by interviewees, spanning the personal, relational, and environmental (Table 1). Certain barriers reflected qualities, behaviours, and feelings of those engaged: lack of knowledge, lack of awareness of engagement in research, cliques, internalised powerlessness, and feeling intimidated. Some barriers reflected qualities and attitudes of researchers: lack of engagement experience, lack of buy-in for engagement practice, playing favourites, and devaluing community knowledge. Other barriers reflected how engagement was organised: funding, lack of diversity, bureaucracy, logistics, technology, time, language, and lack of or insufficient compensation.

**Table 1:** Barriers to power-sharing in health research. *Source*: Author’s analysis of interview data

Barrier	Description
**Personal factors**	
Lack of buy-in for engagement in health research	By researchers: “I think a lot of researchers, it’s quite a burden so then they see it as something that now they’re being pushed to do more and more, they see it as very time consuming. A lot of researchers question its value, especially some of the researchers that I’m in touch with who are doing quite sort of basic scientific research.” (engagement practitioner, UK) By patients/public: “Communities don’t see research as relevant to their lives.” (engagement practitioner, Australia)
Devaluing knowledge of people with lived experience and members of the public	“I was the only patient advocate in a room of thirteen people, and there was a person there representing a fairly high level of healthcare who obviously didn’t feel the need for a patient in the room. And, I felt that animosity, you know. I’m experienced enough to not, it doesn’t bother me and I can deal with it, but I know that it still exists because people at your level for instance, who’ve spent years and years and years getting your qualifications, find it difficult sometimes to accept a patient into the group who obviously doesn’t share your level of skill.” (person with lived experience, UK)
Internalised powerlessness of people with lived experience and members of the public: Don’t see selves as equal to academic researchers	“I guess for me, being really honest about it I always thought I wasn’t a real researcher, like I always had this kind of niggling feeling of they, the university researchers are the real researchers and that’s just really like a confidence thing and an internalised ableism and an internalised elitism but it was a kind of unconscious thing for me is that I’m not a real researcher, I’m just someone here coming in to kind of you know ask questions and help with a few things… And it’s really hard to battle that, even though you know you have people who are so supportive and being no we want you to inform the research and then they’re really bringing you to the table with it.” (person with lived experience, Australia) “They’re the experts you know. We don’t know enough about medicine and medical procedures to question their judgement.” (person with lived experience, UK)
Feeling intimidated due to education and class disparities	“There will be some people who perhaps are not well educated, who would be frightened by being involved in something like that, but I guess you could say that they’re the sort of people that need to be.” (member of the public, UK) “Now most of the people I know with a mental illness they wouldn’t do that, do you know what I mean, it’s the last thing that they’d do…I think they would feel frightened, they would feel intimidated… I think it is the whole the not knowing, the intimidation, the oh is everybody gonna be all dressed up posh and I’m gonna be wearing my you know stinky hoodie that I’ve worn for the last week, do you know what I mean?” (person with lived experience, UK)
Lack of awareness of engagement in health research	“There’s not enough people out in the general public even know that it’s possible to get involved.” (member of the public, UK)
Lack of knowledge about research or lack of engagement experience	By researchers: “Their view of co-design was far more tokenistic than mine; you kind of read the rhetoric around involvement and they go oh yeah, yeah, yeah, we want that but they don’t really know how to do it, and they also don’t really appreciate that they have to give up power to do it.” (engagement practitioner, Australia) By patients/public: “Initially I was rejected only on the grounds that I was starting the patient public involvement journey and the panel, the professor and the other academics or clinicians on the panel felt that at the time I did not have sufficient PPI [patient and public involvement] experience to sit on the panel.” (person with lived experience, UK)
Too much knowledge (scientific or medical) or engagement experience	By patients/public: “There are researchers that only want to work with kind of new patients, the patients that haven’t been involved in patient involvement before… because the rest of us become almost trained in what to look for, so we identify the weaknesses and the strengths and things like that.” (person with lived experience, UK)
Illness	“Patients can’t travel a lot… So our disease limits us as well you know, it’s a barrier to us as well cause some people just don’t have the energy and are very sick and even on dialysis you’re alive but you’re not, you know, you feel crap.” (person with lived experience, Australia)
**Relational factors**	
Playing favourites amongst those engaged	“But then I noticed, and I would pick up was that I would say something exactly the same, and it was not acknowledged, and then the service user would say exactly what I have said in different words, they’d [the researchers] be like that’s a really good idea, oh yes we should put that, and that’s really important and thank you for bringing that up…it’s almost like they’re [the researchers] pampering one and then absolutely disregarding the other one…making a lot of fuss over your colleague, oh would you like coffee, would you like drink, let me get it for you; how was your holiday.. and then just absolutely just not acknowledging you at all. You will start to feel like undervalued and not important and uncomfortable. But… you can’t go to somebody and say you know I’m sorry but I feel belittled and I feel a bit discriminated or I feel victimised.” (person with lived experience, UK)
Cliques amongst those engaged	“I think the worst ones are the ones where they’re almost personal friends of each other, they work together every time they’re on a research project and it can be really, really hard to break in then, like I said get a word in… [It] happens quite a lot locally and ends up with a few people dominating conversations.” (person with lived experience, UK)
**Environmental factors**	
Technology	Technology: “Often city people can get involved in things cause they can go to meetings or they can go to stuff but it’s barriers with rural people because not everybody has the telecommunication or the you know Wi-Fi, I mean certainly in our area a lot of our, our guys have any. They don’t have an iPad, they don’t have Wi-Fi, they don’t have the ability or a phone, you know they have the old home phone still, it’s all they can afford and you know that’s barriers to people actually being involved in research.” (person with lived experience, Australia)
Getting diversity	“A lot of the PPI contributors and patients I work with are retired, in fact the majority are retired, and they’re older people and trying to get diversity and a younger voice or, and it’s a cop out to say, but it’s particularly difficult. But it’s a very educated, very white, very middle class you know area. And that there are obviously you know areas of diversity and lower socioeconomic group and different ethnicities, but the majority of people who we reach, as I say, we need to do better, we need to try harder to reach those sort of other groups.” (engagement practitioner, UK) “Traditionally in the UK the lay representatives, PPI representatives tended to be white, males and often retired. Over the years, white, female have made inroads in patient public involvement work…but on the funding panels we’re still not seeing enough people from different ethnic minorities …we do not have enough Chinese, Pakistani, Muslims.” (person with lived experience, UK)
Bureaucracy	Lengthy application process and numerous selection criteria for patient and public involvement positions: “For me, that’s the kind of example of the kind of cultural imperialism of you know of PPI is you know well you can come and sit on our committee but only if you tick all these boxes.” (engagement practitioner, UK) Selection criteria included: being a health service user, having computer literacy, having a high reading age, having an interest in the subject, committee experience or at least being able to talk to people, and “increasingly they’re asking volunteers about their networks as well which is a really, really hard one because they expect you to have links to charities and hospitals and clinicians and all this kind of thing.” (person with lived experience, UK)
Logistics	Distance: “I suspect there’s a lot of people who might be interested, who would be quite happy to do something more local to them if it was one afternoon or evening in their local GP surgery for example, or in the local primary school. Because it takes a bit of effort even if you can afford you know the bus fare and train fare, even if you, you know you are interested in these sort of things generally, it still takes a bit of effort.” (member of the public, UK)
Time	“It takes a lot of time to do consensus decision-making and to do genuine co-design.” (engagement practitioner, UK) “Timing is really, really hard because most positions are voluntary and held within kind of working hours.” (person with lived experience, UK)
Language	Using scientific jargon and conducting engagement solely in English: “Well I think part of it is language that is used, so the academics and clinicians making sure they don’t use jargon or technical language that a lay person may not understand.” (person with lived experience, UK)
Lack of or insufficient compensation	“Payment in the UK is such a tricky issue. They would have been paid but it would be nowhere near like you know a fulltime wage or anything. They would be paid for the hours that they put in according to the guidelines that are setup by Involve, which is the organisation you may have heard, you know it’s kind of its, basic rate is sort of a hundred and fifty pounds a day.” (engagement practitioner, UK) “When I went to London they, they gave us travel although they only gave us thirty pounds, which doesn’t cover a peak time rail ticket to London, but it was something and they gave us like a voucher as well, you know, you could spend it at the shops.” (member of the public, UK)
Funding	Engagement not funded pre-grant: “I know funding constraints don’t always allow this but what if you even just got in a group of community researchers and then let them direct the project. Like I know that’s really hard because you know you get a grant around a particular topic area but and then kind of that’s already set so there’s like particular parameters around that.” (person with lived experience, Australia) Funding criteria about engagement absent, not enforced, or not weighted strongly: “They may be not always as firm as they might be about how many different stages that PPI involvement happens in, so I think sometimes it’s still possible to get quite a long way through the research and then to think oh I need some PPI involvement, you know, get together a little group, get some comments, make a few changes maybe.” (member of the public, UK) Engagement cut from grant budget of funded project: “PPI is resource that often gets cut from collaborations’ bids.” (engagement practitioner, UK)

Collectively, the three types of foundations addressed many (but not all) of the barriers to power sharing in health research identified by interviewees. Building connections address barriers to sharing power like devaluing community knowledge, lack of buy-in and awareness of engagement in research, and lack of diversity of those engaged. Researcher supports help address lack of knowledge and buy-in for engagement, make those engaged feel less intimidated by researchers, reduce the lack of diversity amongst those engaged, and reduce feelings of internalised powerlessness. For instance, an interviewee remarked that a safe and encouraging space is essential to capture the voices of those who have had:

intense personal experiences of all their power stripped away…I think there is a feeling of powerlessness that those experiences leaves that you bring with you when you come and sit at the table. And so if we genuinely wanna hear those voices I think we have to go the extra mile to make it a safe space and encouraging space for them to feel that their voices have value and they can be heard. (engagement practitioner, Australia)

Barriers not addressed by the identified foundations were:

- Having too much knowledge (scientific or medical) or engagement experience
- Illness
- Funding
- Bureaucracy
- Logistics
- Technology
- Lack of or insufficient compensation
- Playing favourites amongst those engaged
- Cliques amongst those engaged

Barriers only identified by UK interviewees were: bureaucracy, lack of diversity, and having too much clinical or scientific knowledge or engagement experience (Table 1). The latter was spoken about by two interviewees who had been turned down for engagement roles due to having a clinical background and PhD respectively. The interviewee with a PhD noted that she was thankful her degree was in plant science and not a health-related field because “I quite often have to persuade them that I am actually some use because some people think I’m actually a scientist and that’s not what they’re looking for.” The other interviewee remarked that it seems that researchers at times only want to work with members of the public with no engagement experience and/or members of the public with no background in the area of research: “just Joe or Josephine public”. Other times researchers like to have people who have some background in the area under study or are happy with a mix of people. Australian interviewees spoke about class and education disparities as barriers but did not link them to feeling intimidated, whereas several UK interviewees did.

## Discussion

Robust ethical guidance for authentic engagement cannot be developed unless it is informed by the issues and concerns experienced by both researchers *and* their patient, public, and community partners. This paper begins to identify the essential foundations for meaningful engagement in health research from the perspective of people with lived experience and members of the public. It builds an understanding of power sharing in health research from a non-academic researcher viewpoint.

Although the findings reported in this paper speak to shared decision-making in health research generally, they offer insights as to what foundations are thought to be important to achieve it during health research *priority-setting*. It is particularly vital to develop ethical guidance on inclusive research priority-setting given that agenda-setting in health research is typically dominated by academic researchers and funders. Authentic engagement in health research begins with shared decision-making in priority-setting.

The study shows that relational foundations such as forming connections and creating safe spaces will help people with lived experience and members of the public to share vulnerabilities, which provide key insights for identifying research topics and questions to explore. Environmental foundations such as training about funding processes and pre-grant funding mechanisms for engagement will enable people with lived experience and members of the public to participate in grant writing and facilitate their joining projects before research topics and questions are set. Environmental foundations like having a research culture that values the voices of people with lived experience and members of the public in setting research priorities are important too. Personal foundations such as lead researchers who value engagement will help ensure that people with lived experience and members of the public are involved early on.

The value of forming connections, researcher support, and research culture to sharing power in health research priority-setting are key insights generated by this study. Previous research capturing the perspectives of researchers, ethicists, and community organisation staff also identified building trust, personal qualities and skills of researchers and community members, funding, and broader cultural norms as facilitating shared decision-making in health research priority-setting [34]. The wider literature on participatory development and participatory research, however, has identified forming connections, trust, personal qualities of researchers like cultural humility, and supports like training and pairing those engaged with academic researchers as important to achieving inclusion [6, 7, 11, 24, 32, 44]. The qualities and skills of people with lived experience and members of the public necessary to achieve power-sharing are perhaps less reported in those literatures. Norms around valuing different types of knowledge equally have also been identified as essential, especially when participation occurs in science and technology fields [20]. Use of evidence language as the norm and existing hierarchies in science and technology fields means people with lived experience and the public are less likely to have their ideas and views listened to because they are seen as having lower credibility than health professionals [17, 20, 21, 44].

The study provides more evidence that achieving inclusive health research priority-setting depends on work being done by researchers, engagement practitioners, research institutions, and funders in advance of and independent of funding or conducting specific research projects. What their ethical responsibilities to support authentic engagement might look like in light of the study findings is now considered. It is suggested that researchers, engagement practitioners, research institutions, and funders should adopt certain policies and practices. Their specific obligations are proposed below and assigned because their remits make them especially well-placed to build certain foundations. (Please note, however, these obligations do not encompass the *entirety* of researchers, research institutions, engagement practitioners, and funders’ ethical responsibilities in relation to engagement in health research.)

Funders, research institutions, and engagement practitioners should help create a normative environment where engagement in health research, including during priority-setting, is valued. To do so, governments could run national campaigns to build awareness about engagement roles in health research and funders could set up research design services that support engagement during the development of research proposals. Funders could further demonstrate that they value engagement in health research priority-setting by engaging people with lived experience and members of the public when setting their own priorities and on their grant selection panels. Existing examples to drawn on include the James Lind Alliance, Diabetes UK, UK National Institute of Health Research, and the US Patient-Centred Outcomes Research Institute [10, 18, 26, 29]. Research institutions could make meaningful engagement in health research a core promotion and performance review criterion.

Funders and research institutions should also adopt funding policies, grantmaking principles, and funding selection criteria requiring engagement in health research, with engagement as shared decision-making and starting during agenda-setting being weighted more heavily than engagement as consulting and engagement starting late in research projects. Grantmaking principles could give preference to research teams that include people with lived experience and members of the public. Funders should further offer engagement grants to support forming relationships with communities and to build communities’ capacity to be engaged in health research. Patient-Centred Outcomes Research Institute Engagement grants are one example of such practice [30]. Research institutions should establish engagement departments/units to build strong connections with their local communities and to help build researchers’ training in and value of engagement, including during priority-setting. Engagement practitioners should help achieve both aims.

Researchers should, as part of their general practice, lay the groundwork for meaningful engagement by spending time with the communities with whom they conduct research and by cultivating certain skills and qualities within themselves, e.g., openness, good communication. They should form personal connections with people from those communities and support them to participate in particular research projects from the agenda-setting phase onwards by providing training, accommodating needs, creating safe spaces, and making people feel valued. They should be supported to do this by engagement practitioners/managers and departments at their research institutions.

The findings of this study and proposed ethical responsibilities can usefully be compared to key international research ethics guidelines on community engagement such as UNAIDS *Good participatory practice guidelines for HIV prevention trials*, *Recommendations for community involvement in National Institute of Allergy and Infectious Diseases HIV/AIDS clinical trial research*, and the Council for International Organizations of Medical Sciences’ *International ethical guidelines for health-related research involving humans* [8, 25, 41]. The latter largely does not address building foundations for engagement or who is responsible for doing so. The UNAIDS and NIAID guidance documents do discuss building an environmental foundation—researcher support—and some of the personal qualities identified in this paper—researchers’ value of engagement and their communication skills [25, 41]. Researcher support encompasses training, pairing systems, and accommodating varying needs but does not mention creating safe spaces or making people feel valued [25]. The NIAID recommendations even call for giving training on funding mechanisms and priority-setting [25]. However, none of the guidance documents discuss building relational or other environmental foundations before projects. They also do not assign responsibilities to build foundations to research institutions and funders. Only researchers and community representatives (e.g., community advisory board staff) are identified as obligation-bearers.

Additionally, it is important to note that the identified foundations do not address several of the barriers—largely *structural* barriers—described by interviewees in this study. Potentially many of these barriers (bureaucracy, logistics, compensation) could be addressed through better engagement policies at the government, research institution, and/or funding levels. These policies should be developed with people with lived experience and members of the public. They could (amongst other things) streamline application procedures for engagement roles, call for performing engagement locally, set compensation levels that sufficiently cover people’s time and expenses, and mandate ongoing training in facilitating engagement for all research staff. Future research could usefully explore what foundations are needed to overcome structural barriers to engagement and who should be responsible for establishing them.

Finally, UK interviewees identified several barriers that Australian interviewees did not: bureaucracy, lack of diversity, and having too much clinical or scientific knowledge or engagement experience. This could likely reflect a more bureaucratic system of patient and public involvement in the UK as well as engagement in health research being more established in the UK. Future research could gather information from interviewees in other countries, especially low- and middle-income ones, as their voices need to be captured to inform ethical guidance on engagement in health research as well. Additional barriers and foundations within the three (or additional) categories may be identified by such studies.

## Conclusions

This article provides initial evidence about what people with lived experience and members of the public think are essential foundations to build to share decision-making in health research priority-setting and health research more broadly. Capturing their perspectives is necessary because they offer key insights that will otherwise be excluded, which is a form of epistemic injustice.

Importantly, interviewees identified relational and environmental foundations in addition to individual level personal foundations. Researchers, supported by engagement practitioners/managers, research institutions, and research funders are well positioned to create these foundations. Their policy and practice should thus encompass building them to support the meaningful engagement of people with lived experience and members of the public in health research priority-setting.

## Data Availability

The de-identified datasets used and/or analysed during the current study are available from the corresponding author on reasonable request.

## Abbreviations

UK: United Kingdom
CIOMS: Council for international organizations of medical sciences
NIAID: National institute of allergy and infectious diseases (US National Institutes of Health)
UNAID: Joint United Nations programme on HIV/AIDS

## Footnotes

1 They use the service being researched, have the condition being researched, or are from the community being researched.

2 The paper’s focus is not meant to imply it is sufficient to share decision-making in the priority- setting phase of research alone. Engagement is critical in all phases of health research projects.

3 Within UK policy, public participation has been presented as a solution to help renew and improve society’s trust, interest and relationship with science since 2004. Many UK funding bodies ask that researchers include patient and public engagement in their research plans. Over the last 15 years, UK research funders have embarked on several large-scale collaborative initiatives to support patient and public engagement within research [42].

4 Research4Me is an extended network of people and organisations in Australia that share a common passion for involving people in health and medical research: https://research4.me/

5 This is despite recruitment scripts asking for such participants.

6 According to a person with lived experience from Australia, credibility means being trusted and believed in to provide useful information that is “maybe even essential” to what the researchers are going to do. Developing credibility takes substantial time and entails convincing researchers that the person being engaged is interested in what they do and in seeing it succeed and that they can help shape the research in ways that will make it more beneficial.
